# Age, period and cohort effects on suicide mortality in Russia, 1956−2005

**DOI:** 10.1186/s12889-017-4158-2

**Published:** 2017-03-07

**Authors:** Tanya Jukkala, Andrew Stickley, Ilkka Henrik Mäkinen, Aleksei Baburin, Pär Sparén

**Affiliations:** 1 Stockholm Centre for Health and Social Change (SCOHOST), Södertörn University, Huddinge, Sweden; 2 Department of Sociology, Uppsala University, Uppsala, Sweden; 3 Department of Epidemiology and Biostatistics, The National Institute for Health Development, Tallinn, Estonia; 4 Department of Medical Epidemiology and Biostatistics, Karolinska Institutet, Stockholm, Sweden

**Keywords:** Suicide, Russia, Age-period-cohort analysis

## Abstract

**Background:**

Russian suicide mortality rates changed rapidly over the second half of the twentieth century. This study attempts to differentiate between underlying period and cohort effects in relation to the changes in suicide mortality in Russia between 1956 and 2005.

**Methods:**

Sex- and age-specific suicide mortality data were analyzed using an age-period-cohort (APC) approach. Descriptive analyses and APC modeling with log-linear Poisson regression were performed.

**Results:**

Strong period effects were observed for the years during and after Gorbachev’s political reforms (including the anti-alcohol campaign) and for those following the break-up of the Soviet Union. After mutual adjustment, the cohort- and period-specific relative risk estimates for suicide revealed differing underlying processes. While the estimated period effects had an overall positive trend, cohort-specific developments indicated a positive trend for the male cohorts born between 1891 and 1931 and for the female cohorts born between 1891 and 1911, but a negative trend for subsequent cohorts.

**Conclusions:**

Our results indicate that the specific life experiences of cohorts may be important for variations in suicide mortality across time, in addition to more immediate effects of changes in the social environment.

## Background

Russia has one of the highest suicide mortality rates in the world, 19.7 suicides per 100 000 inhabitants in 2011 [1]. Moreover, Russian suicide mortality has changed rapidly over the past 50 years. A stable increase from the mid-1960s was followed by a near 40% decrease between 1984 and 1986. A slow increase from 1986 was followed by a rapid 60% increase between 1991 and 1994 [2]. These changes in suicide mortality have been associated with Mikhail Gorbachev’s reforms (*perestroika*, *glasnost*) during the 1980s [3, 4] – the anti-alcohol campaign in particular [5, 6] – and with the collapse of the Soviet Union in 1991 and its immediate consequences, such as societal *anomie*, i.e. a state of normlessness, [7–9], steep and prolonged declines in GDP [10], increased levels of stress [7, 11], as well as increased alcohol consumption [6]. Since 1995, the suicide rate has generally been decreasing, the average yearly decrease being 2.8% [12].

Changes in Russian suicide mortality rates since the 1960s have thus been studied as *period effects,* that is, associated with changes in the social environment occurring *at the same time* as the changes in suicide [3–12]. However, societal events might also have long-lasting effects on the suicide patterns of a generation of individuals exposed to similar factors during their childhood and early adult life. It is thus important to assess such *cohort effects* [13] that possibly constitute part of the explanation behind the changes in Russian suicide mortality. To the best of our knowledge, no previous studies have examined cohort effects in the context of Russian suicide mortality.

Age, period and cohort (APC) analysis can discern between period and cohort effects in terms of the changes in e.g. suicide mortality rates, while also taking into account variations according to age. Previous studies that have used descriptive APC analysis to examine changes in suicide mortality rates have reported that cohort effects underpin the increasing suicide mortality seen among younger age groups [14–21]. However, as many of these studies were not able to follow cohorts over the larger part of their life course, the underlying assumption has been that cohorts with a higher suicide risk at lower ages would also have an increased suicide mortality risk at every older age. But, this assumption is actually contradicted by the results from studies which have indicated a lack of fixed developments in suicide mortality over the life course of a cohort [22–26] and that have instead emphasized the changing nature of cohort-specific developments in suicide in relation to changes in the social environment.

By considering such complexity, APC modeling allows cohort effects to be discerned more effectively from those relating to the period as well as to the age distribution of suicide. Results from such analyses are nevertheless inconclusive regarding the importance of cohort effects as opposed to period effects. While some studies have found cohort effects to be of little importance for changes in suicide mortality compared to period effects [27–29] others have found underlying period *and* cohort effects in the changes in suicide mortality [30–35]. Chung et al [13] found that cohort effects were more important than period effects for the changes in suicide mortality in Hong Kong during the 1976 − 2010 period. The varying importance attributed to cohort and period effects in previous research might, to some extent, reflect the use of different analytical methods, as well as variations in the way results have been interpreted. However, it is possible that period and cohort effects, or the immediate and more long-term effects of societal events and processes, might also vary across social contexts and over time [25].

The aim of the current study was to examine period and cohort effects behind the changes in Russian suicide mortality between 1956 and 2005 by using descriptive analyses and APC modeling. As previous research has indicated that the importance of period and cohort effects can differ between the sexes [13], the analyses were conducted separately for men and women.

## Methods

### Data

Seen as politically sensitive, suicide mortality statistics (together with those on cholera, plague, homicide, and occupational accidents), were kept confidential in the former Soviet Union until the late 1980s [36]. By undertaking archival work Russian and French demographers were later able to retrieve and reconstruct a continuous series of age- and sex-specific suicide mortality data for the Russian population from 1956 to 1989 from these previously confidential records [37]. The present study uses this data as well as age- and sex-specific suicide data for the Russian population from the Russian State Statistical Service (*Rosstat*) from 1990 onwards. The population figures for the years 1956 − 2005 came from *Rosstat* and its predecessor *Goskomstat*.

### Analysis

Descriptive analyses and APC modeling were used to examine suicide mortality rates calculated as averages for 14 five-year age groups (15 − 19, …, 80 − 84) during 10 five-year periods (1956 − 1960, …, 2001 − 2005). Data for the 85+ age group were excluded due to our inability to be able to designate this age group to any specific cohort. Cohorts were constructed by identifying the birth years of the population represented in the available suicide data; for example, those who were aged 15 − 19 in 1956 − 1960 would belong to the cohort born between 1937 and 1945. Consequently, 23 partially overlapping cohorts were constructed (1872 − 1880, 1877 − 1885, …, 1982 − 1990). Age-specific suicide rates were then tracked forward for each cohort; for example, to calculate the 20 − 24 age-specific rate for the cohort born between 1937 and 1945 the mean suicide rate of the 20 − 24 age group over the years of 1961 − 1965 was used. The different cohorts are referred to in terms of the year that constitutes their mid-point (for example 1941 for the 1937 − 1945 cohort).

Regression models were fitted to the data based on the assumption that the number of cases followed a Poisson distribution. The effects of age, period and cohort on the dependent variable were assumed to be multiplicative, and the parameters were estimated with the maximum likelihood method. Although the model was overdispersed due to the large number of deaths, the assumption of a Poisson distribution was retained in order to comply with common practice in APC modeling. However, F-tests were used instead of tests based on the Chi-square distribution to account for this overdispersion when testing different models. In a sensitivity analysis, the data were re-analyzed utilizing a negative binomial regression model, which is usually preferred for overdispersed count data [38]. Since the results from the two models were generally similar, and did not alter our interpretation of the data, only results from the Poisson regression model are presented below.

Linear dependency between the age, period and cohort components, (i.e. cohort = period – age) [39] is a fundamental problem of all APC models, making it impossible to estimate all three effects simultaneously in the full age-period-cohort model [40]. Separate models were therefore constructed where the effect of age was assumed to be constant, while period and cohort effects were allowed to vary freely. Models with both linear and curvilinear approximations of the trends in the age structure were constructed as initial analyses of the data showed that, for men in particular, the age structure of a given period was curvilinear rather than linear. With age held constant, unique regression parameters for the period and cohort effects could be obtained.

The parameters represent the relative risk for suicide in relation to the different periods and cohorts compared to a reference group (relative risk = 1). The direction of the parameter estimates indicates an increase (upwards slope) or decrease (downwards slope) in the relative risk across periods/cohorts, while a stationary slope indicates stability.

APC modeling was conducted by using SAS version 9.4 procedure PROC GENMOD.

## Results

### Descriptive statistics

**Fig. 1:**
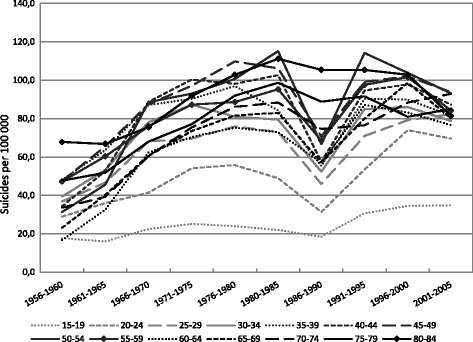
Period specific suicide rates (per 100 000) for Russian males by age group

Age-specific trends in male suicide mortality between 1956 and 2005 are shown in Fig. 1. A generally greater increase in suicide for every successive male age group up to the mid-1980s is reflected in an increased difference in the level of suicide between the youngest and the oldest age groups. From the mid-1990s a reverse trend can be seen as suicide mortality generally decreased more rapidly for every successive age group and even increased among the youngest age groups (15 − 29). The level of suicide among the age groups between 35 and 54 diverges from the general trend over the period 1956 − 2005 in terms of being consistently high and sometimes even exceeding that of older age groups. Moreover, there was a decrease in suicide mortality in most male age groups in the five-year period during and after Gorbachev’s political reforms (including the anti-alcohol campaign) (1986 − 1990) and an increase in the five-year period following the break-up of the Soviet Union (1991 − 1995).

**Fig. 2:**
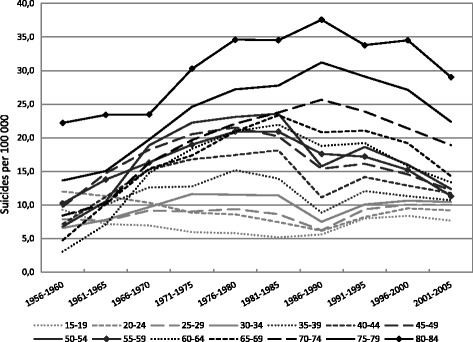
Period specific suicide rates (per 100 000) for Russian females by age group

Similar trends in suicide mortality in the period between 1956 and 2005 can be seen among women (Fig. 2), i.e. a generally greater increase in suicide for every successive female age group up to the mid-1980s, a generally greater decrease in suicide for every successive age group from the mid-1990s, and even an increase in suicide mortality for the youngest (15 − 34) age groups. Compared to men, however, there is a more consistent pattern of a higher suicide rate for every older age group among women. The changes in the periods 1986 − 1990 and 1991 − 1995 were less marked among the female age groups, than among males.

**Fig. 3:**
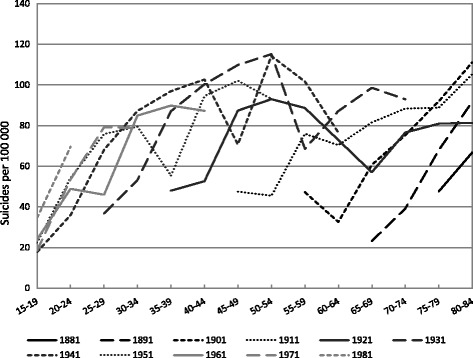
Age-specific suicide rates (per 100 000) among Russian males by birth cohort

**Fig. 4:**
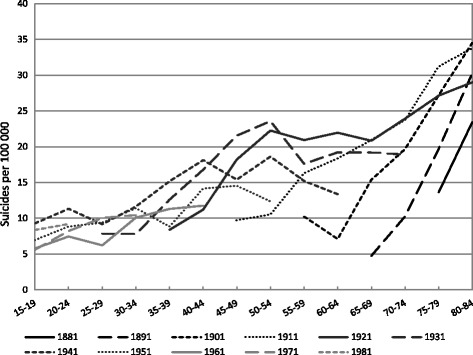
Age-specific suicide rates (per 100 000) among Russian females by birth cohort

Age-specific variations in suicide mortality for the male and female cohorts are shown in Figs. 3 and 4, respectively. For a better overview only every other cohort is presented in the figures. The general decrease in suicide mortality in 1986 − 1990 and increase in 1991 − 1995 are also discernible in the cohort-specific trends in male suicide mortality (for example, in the ages 55 − 59 and 60 − 64 for the 1931 cohort) (Fig. 3). Among the male cohorts born up until 1931, suicide mortality rates tend to be higher at every age for every younger cohort. Among the cohorts born after 1931 such cohort effects are more difficult to discern due to the large periodic variation. However, the level of suicide mortality is higher at young ages (15 − 29) among the cohorts born from 1971 onwards.

The general decrease in suicide mortality in the five-year period 1986 − 1990 and increase in 1991 − 1995 is similarly discernible among female cohorts (Fig. 4). Among the female cohorts born up until 1921 suicide mortality rates tend to be higher at all ages for every younger cohort. Among the cohorts born after 1921 such cohort effects are difficult to discern due to the variation in suicide rates in the periods between 1986 and 1995. The cohorts born after 1971 do however have a higher level of suicide at young ages (15 − 29).

### APC modeling

As expected, the full age-period-cohort model had the best fit among men (df 96; Deviance 5260) and women (df 96; Deviance 6321) (Table 1). Moreover, the age-period model was better fitted than the age-cohort model among men and women, although for women, the difference between the models was less marked. For men a superior model was achieved with a curvilinear trend for age compared to a linear trend (*p* < 0.0001), while for females there was no significant difference between the two models (*p* = 0.67).

**Table 1:** Results of fitting Poisson regression models to suicide mortality data for men and women in Russia

Model	Men			Women		
	df	Deviance^a^	*P*-value^b^	df	Deviance^a^	*P*-value^b^
Age	126	92914		126	21822	
Age + drift	125	64573		125	21073	
Age + period	117	14233	<0.0001^c^	117	12062	<0.0001^c^
Age + cohort	104	53555	<0.0001^c^	104	14452	<0.0001^c^
Age + period + cohort	96	5260		96	6321	
Linear age + period + cohort	108	85928	<0.0001^d^	108	10986	0.67^d^
Curvilinear age + period + cohort	107	38892		107	10968	

*df* Degrees of freedom

^a^Deviance from the Poisson model

^b^ *P*-value based on a test with F-statistic

^c^Compares the partial model with the full age-period-cohort model

^d^Compares the linear model for age with a curvilinear model

**Fig. 5:**
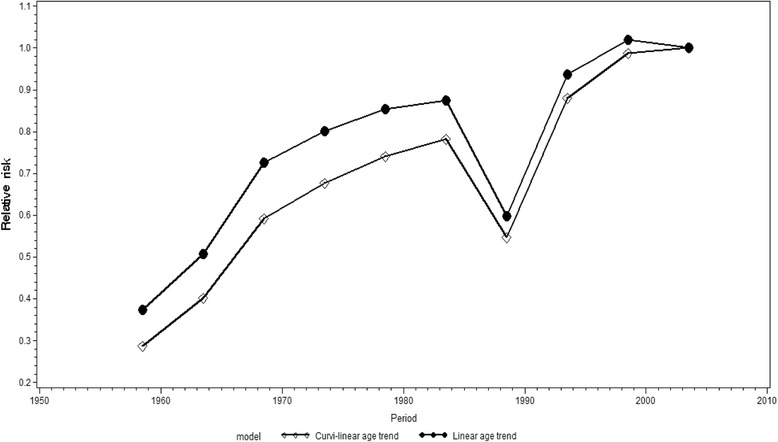
Period-specific changes in the suicide risk for males in Russia, 1956 − 2005. Estimates are calculated by an APC model, with curvilinear and linear approximations of age structure (assumed constant). (Reference group = period 2001 − 2005)

The period-specific risk for male suicide increased almost constantly, with the exception of the 1986 − 1990 period when it decreased, and in 2001 − 2005, when it was more or less stable (Fig. 5). The steepest increase in the period-specific relative risk for suicide was observed in the 1991 − 1995 period, while the increase in the 1966 − 1970 period although less sharp, nevertheless stood out from the overall trend. When the age effect was held constant with a curvilinear approximation, the increase in the relative risk over the whole period was somewhat greater.

**Fig. 6:**
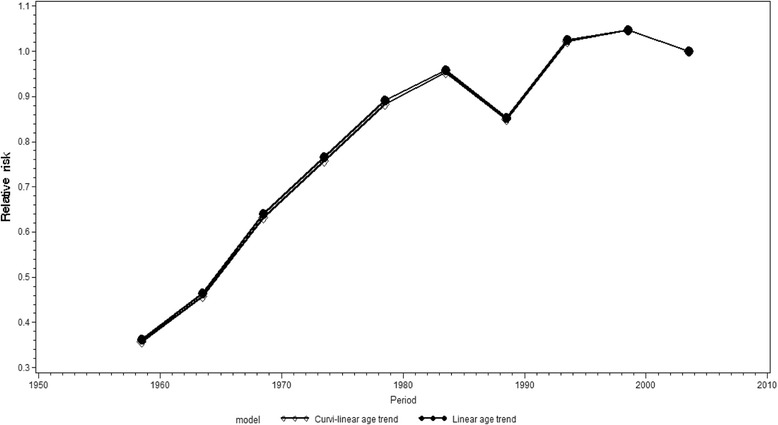
Period-specific changes in the suicide risk for females in Russia, 1956 − 2005. Estimates are calculated by an APC model, with curvilinear and linear approximations of age structure (assumed constant). (Reference group = period 2001 − 2005)

The trends in the period-specific suicide risk for women were rather similar to those of men, although both the decrease in the period-specific relative risk in 1986 − 1990 and its increase in the following period (1991 − 1995) were smaller than those observed for men (Fig. 6). Moreover, among women the increase in the period-specific relative risk for suicide was more constant up until the 1981 − 1985 period. The models with curvilinear and linear age structure approximations had identical results.

**Fig. 7:**
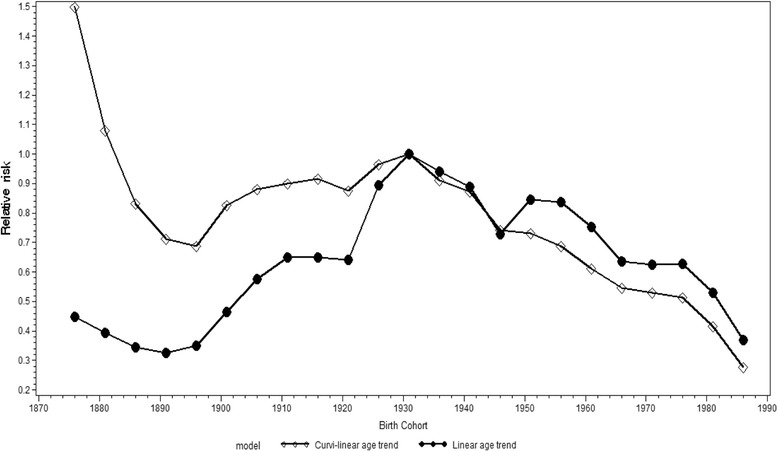
Cohort-specific relative risks for suicide among males in Russia, 1956 − 2005. Estimates are calculated by an APC model with curvilinear and linear approximations of age structure (assumed constant). (Reference group = 1931 cohort)

Male cohort-specific relative risk estimates for suicide decreased for the four oldest cohorts (born between 1876 and 1891), increased among the cohorts born between 1896 and 1931, and decreased among the cohorts born from 1936 onwards (with the exception of the cohort born in 1951) (Fig. 7). The changes in the relative risk estimates of the oldest and youngest cohorts should however, be interpreted with some caution as there are relatively few observations for them. The models with curvilinear and linear approximations of age produced differing cohort-specific effects, especially for the older cohorts. However, the trends were similar.

**Fig. 8:**
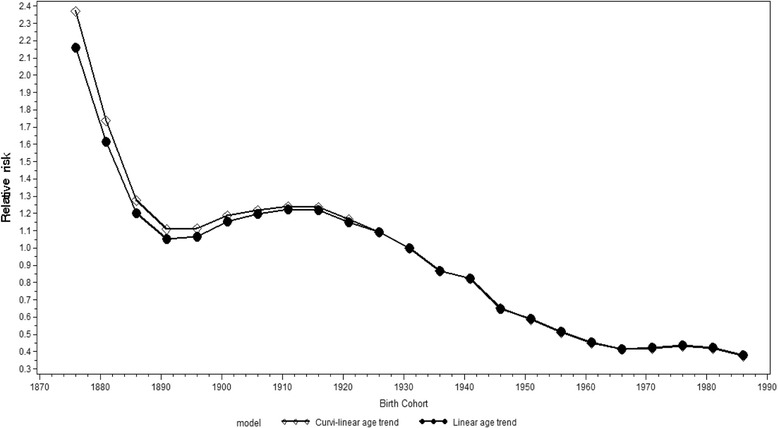
Cohort-specific relative risks for suicide among females in Russia, 1956 − 2005. Estimates are calculated by an APC model with curvilinear and linear approximations of age structure (assumed constant). (Reference group = 1931 cohort)

Female cohort-specific relative risk estimates for suicide followed a similar trend to those for males. There was a decreasing trend in the relative risk among the four oldest cohorts (born between 1876 and 1891). This was followed by a weak positive trend for the cohorts born from 1896 onwards, but only up to the 1911 cohort (Fig. 8). Moreover, the decreasing trend in the cohort-specific relative risk occurred earlier among females, in the cohorts born from 1921 onwards, and stabilized in the cohorts born from 1971 onwards. It should be borne in mind, however, that as with men, there were few observations for either the oldest or youngest cohorts.

## Discussion

### Age specific trends

Descriptive analyses revealed both change and stability in the age structure of suicide over the period. Across the period as a whole there was a relatively stable pattern of increasing rates for every older age group. However, there was a greater increase in suicide rates for every older age group in the period between 1956 − 1985 followed by a greater decrease in suicide for every older age group, and even an increase among the youngest age groups (15 − 34), in the period between 1996 − 2005. The changes in the latter period raise concerns for the youngest in Russia.

A recent report also noted an increase in youth suicide in Russia in the 2002 − 2009 period and moreover, that suicide among Russian youth was comparatively high in international terms [41]. It is possible that changes in societal conditions from the mid-1990s onwards have been especially detrimental for younger persons. For example, young Russians report a range of differing worries that touch on such things as the lack of employment opportunities and feelings of insecurity about finding a job that matches their skills [42]. Indeed, similar relative increases in suicide among the youngest age groups compared to older ones have also been observed in a number of industrial countries over the latter half of the twentieth century [43–46]. The changes in Russia may thus also be reflecting this wider development.

In contrast to the relatively greater increase in suicide mortality observed among the younger age groups in the period between 1996 − 2005 there was a relatively greater decrease in suicide mortality for every older age group, leaving the oldest age groups with a relative advantage. Gavrilova et al. [11] noted similar developments during the 1990s and suggested that despite their small pensions elderly Russians often had to support their older children financially as a result of high levels of unemployment or unpaid wages. This may have possibly increased their social status and self-esteem and consequently protected them from suicide. A study by Innamorati et al. [47] also showed that the suicide risk for elderly Russian men (65+) compared to other age groups within the country was lower than that for elderly men compared with younger age groups in the EU. On the other hand, the risk of suicide among elderly men in Russia was about double that of men of the same age in the EU countries.

The suicide rate among middle aged males (30 − 54-year-olds) was notable for being consistently high over the period 1956 − 2005, as has been previously reported [2]. One possible explanation for this could be the particularly high frequency of alcohol consumption among males in this age range [48]. When examining the relationship between alcohol consumption and suicide in Russia, Razvodovsky [49] also found it to be strongest among men of this age.

### Period effects

The decrease and increase in suicide mortality rates in the 1986 − 1990 and 1991 − 1995 time periods, respectively, was clearly discernible in most male and female age groups and cohorts in the descriptive analyses. These results emphasize the importance of period effects in relation to Russian suicide, at least as regards the particular years during and immediately after Gorbachev’s *perestroika* reforms and the fall of the Soviet Union. As shown by previous research, these huge fluctuations in Russian mortality rates, including suicide mortality, were strongly related to the changes in alcohol consumption as a result of the anti-alcohol campaign in the 1980s and the liberalization of alcohol market in the 1990s, that was accompanied by increased illegal production of cheap and often highly toxic alcoholic beverages [6, 50].

Estimates of the period-specific relative risks for suicide mortality from the APC models further indicated that period effects were not limited to 1986 − 1990 and 1991 − 1995. More specifically, apart from decreases in 1986 − 1990 and 2001 − 2005, period-specific relative risk estimates increased continuously. Thus, societal events and processes that have influenced suicide mortality in Russia seem to have had a mainly elevating effect on it.

### Cohort effects

Both the descriptive analyses and the APC-models indicated an increased suicide mortality rate for every subsequent cohort among the older male and female cohorts. The cohort-specific relative risk estimates revealed an increasing trend for the male cohorts born between 1896 and 1931 and for the female cohorts born between 1896 and 1911. These cohorts experienced both life in traditional Russian society and the processes of modernization (i.e. industrialization, urbanization, secularization) that started slowly during the last decades of the nineteenth century and continued, after a pause, at a particularly rapid pace in the late 1920s [51, 52]. Durkheim [53] argued that modernization leads to insufficient levels of societal integration and regulation of goals and needs, which results in an increase in *egoistic* and *anomic* suicides. Societal modernization has also been associated with increasing suicide rates in the West [33, 44, 54, 55].

In addition, there was an increase in the relative risk of suicide among the male cohorts who experienced their youth during the onset of Stalin’s dictatorship (1922 − 1953), a period of political terror, harsh agrarian reforms, famines and great human losses during World War II. In contrast, women who spent their youth in that political environment did not have increased cohort-wise risks later in their lives. This might indicate the greater sensitivity of male suicide mortality to social factors, and parallels the greater increase seen in male compared to female suicide mortality, during the period (1991 − 1995) following the break-up of the Soviet Union.

The male generations born after 1931 and the female generations born after 1911 showed a decreasing trend in suicide mortality. This contrasts with the general increase in suicide mortality over the period 1956-2005. A similar finding was also observed in a study of cohort effects in Lithuanian suicide mortality during the 1970 − 1995 period where there was a decreasing trend in the cohort-specific suicide risk among male cohorts born between 1950 and 1965, and female cohorts born between 1925 and 1970, despite an overall increase in the level of suicide mortality [34].

The increasingly lower cohort-specific suicide risk among the male generations born after 1931 and the female generations born after 1911 could be related to the fact that these cohorts grew up while modernization was occurring in modern Russia. They might thus have been better adapted to life in modern society (even in its particular, Russian version), compared to older generations, and perhaps even enjoyed some of the benefits of a modern lifestyle. This would accord with Halbwachs’ [56] notion that the initially elevating effects of modernization on suicide would be interrupted once societies became accustomed to modern life (i.e. as was the case in some Western European countries during the latter half of the twentieth century). Further support for this idea has been found in later studies from the West [35, 44, 54, 55]. Such developments might possibly be associated with a generational shift towards a lower cohort-related risk for suicide.

The cohort specific risk estimates revealed no increase in suicide rates among the youngest cohorts, indicating that the changes in the age structure of suicide, where there was a relative increase in suicide among the younger ages, was not due to a cohort effect. There are however, still too few suicide deaths among younger cohorts from which to properly determine cohort-specific developments in suicide mortality.

### Limitations

Data on violent mortality from the Soviet period are generally considered as being reliable [57]. However, there may have been some deterioration in data quality during the post-Soviet period [58, 59].

Moreover, the periodic data used in the present study only allow for approximations of cohorts with a certain degree of overlap between them, thus, they do not represent real birth cohorts, i.e. constituting an exact group of people born in a specific period which can be followed over their life course. The study also had no access to data covering the younger ages of the older cohorts or of the older ages of the younger cohorts, which means that for those groups the analysis was only based on partial data. This, however, is not unusual for this type of analysis.

Another limitation is the exclusion of the 85+ male and female age groups from the APC analyses. This was necessary due to the fact that cohorts could not be specified for this age group. We are unable to assess how the exclusion of this age group may have affected the estimates of age, period and cohort effects respectively.

In order to separate age, period, and cohort parameters this study assumed that the effect of age was constant (with linear as well as curvilinear approximations). Despite certain variations in the age structure across time, this assumption is seen as valid considering the generally stable pattern of increasing suicide mortality with increasing age.

## Conclusions

To the best of our knowledge, this is the first study of suicide mortality in Russia to examine both period and cohort specific effects. Two different processes seem to have been working counter to each other in relation to the changes in suicide mortality during the period 1956 − 2005. On the one hand, the immediate effects of events and processes in Russian society over the period have caused suicide mortality rates to generally increase. On the other hand, the lower cohort-related suicide risk among younger male and female cohorts (born after 1931 and 1911 respectively) indicates long-term effects on the suicidal patterns of these cohorts resulting from exposure to similar factors during their development and early adult life. Societal events, changes and processes may thus have both immediate and long-term effects on suicide mortality, which may be working in different directions in a given period. This highlights the potential importance of understanding both period and cohort effects in relation to changes in suicide mortality over longer time periods.

## Abbreviation

APC analysis: Age-period-cohort analysis
